# Influence of Measurement Geometry and Blank on Absolute Measurements of Photoluminescence Quantum Yields of Scattering Luminescent Films

**DOI:** 10.1021/acs.analchem.4c06726

**Published:** 2025-03-07

**Authors:** Florian Frenzel, Saskia Fiedler, Ahmad Bardan, Arne Güttler, Christian Würth, Ute Resch-Genger

**Affiliations:** Division Biophotonics, Federal Institute for Materials Research and Testing (BAM), Richard-Willstaetter-Strasse 11, Berlin D-12489, Germany

## Abstract

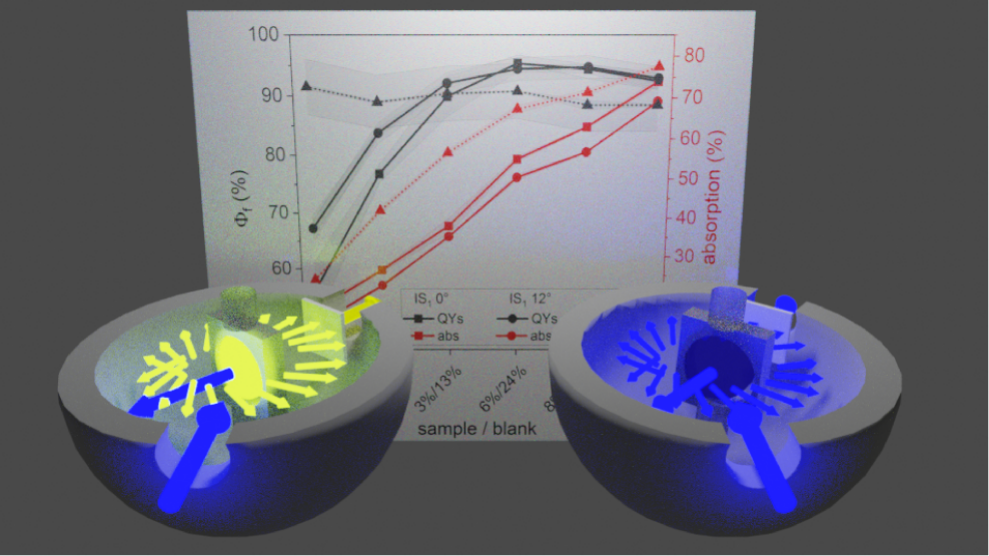

For a series of 500 μm-thick polyurethane films containing different concentrations of luminescent and scattering YAG:Ce microparticles, we systematically explored and quantified pitfalls of absolute measurements of photoluminescence quantum yields (Φ_f_) for often employed integrating sphere (IS) geometries, where the sample is placed either on a sample holder at the bottom of the IS surface or mounted in the IS center. Thereby, the influence of detection and illumination geometry and sample position was examined using blanks with various scattering properties for measuring the number of photons absorbed by the sample. Our results reveal that (i) setup configurations where the scattering sample is mounted in the IS center and (ii) transparent blanks can introduce systematic errors in absolute Φ_f_ measurements. For strongly scattering, luminescent samples, this can result in either an under- or overestimation of the absorbed photon flux and hence an under- or overestimation of Φ_f_. The size of these uncertainties depends on the scattering properties of the sample and instrument parameters, such as sample position, IS size, wavelength-dependent reflectivity of the IS surface coating, and port configuration. For accurate and reliable absolute Φ_f_ measurements, we recommend (i) a blank with scattering properties closely matching those of the sample to realize similar distributions of the diffusely scattered excitation photons within the IS, and (ii) a sufficiently high sample absorption at the excitation wavelength. For IS setups with center-mounted samples, measurement geometries should be utilized that prevent the loss of excitation photons by reflections from the sample out of the IS.

## Introduction

Photoluminescent materials are broadly utilized in the life sciences and in photonic technologies, with applications ranging from sensing and bioimaging to barcoding, energy conversion, solid-state lighting, and display technologies.^1−14^ All these applications require the characterization of the luminophores’ fundamental and application-relevant properties.^1,15−18^ This includes the spectral positions of the absorption and emission bands, their spectral widths and overlap, as well as quantities providing a measure for the efficiency of the absorption and emission processes, like the molar absorption coefficient or absorption cross-section and the photoluminescence or fluorescence quantum yield (Φ_f_).^9,19^ Φ_f_ is defined as the ratio of the number of emitted to absorbed photons and describes the efficiency of converting absorbed photons into emitted photons.^20,21^ As most applications, specifically those involving energy conversion such as solid-state lighting, displays, light-emitting diodes (LEDs), and solar concentrators, require highly efficient luminescent materials, the determination of Φ_f_ presents a key measurement for photophysical studies, material selection, and material design, i.e., the tailoring of the next generation of functional luminophores. Φ_f_ of transparent samples, such as dye solutions or dispersions of semiconductor quantum dots with sizes of <10 nm, can be determined relative to a fluorescence Φ_f_ standard of reliably known and preferably certified Φ_f_ values using a conventional photometer and fluorescence spectrometer. Scattering samples, like dispersions of larger luminescent nanoparticles or microparticles, solid phosphors, particles, or phosphor powder embedded in films, require absolute measurements of Φ_f_, e.g., with an integrating sphere (IS) setup.^22,23^ Such setups are commercially available as stand-alone devices and as IS accessories for fluorescence spectrometers. These setups and accessories are available in different geometric configurations, with most of them offering the possibility to mount the sample in the IS center. Some offer the possibility to place the sample in and out of the excitation beam, thereby enabling direct and indirect sample illumination.^23−25^ For devices designed for the usage of cuvettes for measuring liquid samples, a center-mounted sample position can be regarded as a standard geometry. For solid samples or powders with different shapes, some manufacturers offer a configuration in which the sample lies on the surface of a sample holder, which is placed on the IS surface.^26,27^

The scientific and industrial importance of reliable Φ_f_ data for the comparison of nanoscale and molecular luminophores and the design of functional luminophores with improved performance has, meanwhile, triggered a critical evaluation of Φ_f_ standards recommended in the literature^21,28−30^ for relative Φ_f_ measurements of transparent samples. This has also led to the development of protocols for relative and absolute Φ_f_ measurements, presently with a focus on transparent luminescent samples.^22,31−35^ However, despite the increasing relevance of scattering solid samples such as solid phosphors, semiconductor quantum dots, or perovskites embedded in a polymer matrix or optoceramics for energy conversion, solid-state lighting, or nanophotonics, the reliable determination of Φ_f_ of such scattering luminescent samples is still challenging, and sources of uncertainty have been rarely quantified.^36−38^ In addition to accurately considering the scattering properties of such samples, the reliable positioning and mounting of such samples inside an IS can present a challenge, e.g., for films, requiring specialized equipment such as specific sample holders and mounting tools.

As an extension of our recently performed interlaboratory comparison (ILC) on absolute Φ_f_ measurements of scattering particle dispersions and solid optoceramics used as LED converter materials with three commercial IS setups with fixed measurement geometries,^36^ in this study, we focus on exploring possible pitfalls and achievable uncertainties for Φ_f_ measurements of scattering luminescent films with common illumination and detection geometries and different blanks. For these measurement geometries, we systematically explore the influence of the sample position in the IS and the optical properties of the blank on the resulting Φ_f_ values. To cover all measurement geometries broadly utilized by IS setups, for the Φ_f_ measurements, two IS setups were employed: (i) a commercial stand-alone IS setup with a fixed measurement geometry, utilized in a recently published ILC,^36^ and (ii) a versatile, custom-made research IS setup, used, e.g., for certifying dye solution-based Φ_f_ standards in 2022/2023.^30^ The latter is equipped with a custom-designed rotatable cuvette holder for precise angle-dependent positioning of samples mounted on the IS center. This configuration enables the flexible choice of the illumination and detection geometry and provides precise control of the angle of light exciting or illuminating the sample. In this study, we focus on the two limiting cases of the previously screened excitation geometry where the excitation light is either back reflected from the sample surface and (i) leaves the IS (θ = 0°) or (ii) remains in the IS (θ = 12°). θ represents the angle between the *k* component of the excitation light and the surface normal of the sample. The angles in between and beyond the limits, i.e., θ = 0° and 12°, were previously screened to identify limiting cases where the excitation light either leaves or completely remains in the IS. The chosen θ values are specific to the custom-built setup used, but the underlying physical principles evaluated are universally adaptable to other home-built and commercial setups, regardless of a fixed or an adjustable angle of incidence. As exemplary scattering films, we prepared a series of 500 μm-thick polyurethane films that were either unstained, stained with varying amounts of scattering luminescent YAG:Ce phosphor microparticles, or nonemissive BaSO_4_ microparticles. To calculate the number of absorbed photons from the incident excitation light flux, (i) transparent nonemissive polyurethane films and (ii) polyurethane films containing different amounts of BaSO_4_ scatterers were employed as blanks. The results of this study allowed us to identify and quantify sources of uncertainty in Φ_f_ measurements of scattering luminescent films and to derive optimum conditions for accurate and reliable Φ_f_ determination of scattering luminescent samples.

## Experimental Section

### Materials and Methods

Polyurethane VT 3402 KK (Wepuran casting resin), hardener H44, accelerating agent B 4402, and adhesion promoter AP 4 LED were purchased from Lackwerke Peters GmbH. MP1300 Micropowder Art. Nr. 200132 and 200440 was obtained from Dolder-Bigler AG, and the film applicator (PG-024–03) from Thierry Präzisionslackiertechnik GmbH. An IKA Roller 10 digital instrument was used for homogenization.

### Sample Preparation

For the preparation of the polymer films homogeneously stained with scattering YAG:Ce microparticles, mimicking common LED converter materials, a defined amount of the YAG:Ce powder was added to a polyurethane hardener mixture (1:1 ratio) and rolled for 45 min to obtain a homogeneous dispersion. For films with a high YAG:Ce concentration, polyurethane and YAG:Ce powder were mixed prior to the hardener addition. Before film casting, the carrier foil was thoroughly cleaned. The film applicator was adjusted to the desired thickness of 500 μm and set to the lowest speed to prevent bubble formation in the resulting films. After casting, the films were cured for about 24 h at room temperature (22 ± 2 °C). Then, the YAG:Ce-containing polymer films were carefully separated from the carrier foil and cut into small circles fitting into the cuvette used for the Φ_f_ measurements with both IS setups.

### Instrumentation

Absolute Φ_f_ measurements were performed with two independently calibrated IS setups, IS_1_ and IS_2_, which differ in sample illumination and detection geometry, as well as in sample positioning within the IS.

#### IS Setup IS_1_

The custom-built IS setup IS_1_ consists of a Spectralon-coated IS with a diameter of 15 cm and six ports. A 150 W xenon lamp and a monochromator serve as the excitation light source, which is focused onto the sample. The scattered, absorbed, and emitted photons are detected with a fiber-coupled Shamrock 303i imaging spectrograph combined with a Peltier-cooled deep depletion CCD. Apertures in front of the entrance port of the IS allow for adjusting the excitation spot size on the sample. Baffles within the IS block the detection port in such a way that exclusively diffuse radiation is detected. For the determination of Φ_f_, the blank and sample are mounted in the IS center using a custom-designed 360°-rotatable sample holder equipped with a transparent PMMA adapter. This allows round thin film quartz cuvettes to be fitted into the IS sample holder initially designed for 10 mm × 10 mm cuvettes. For the Φ_f_ measurements, YAG:Ce-containing and nonluminescent transparent or scattering films used as blanks were placed between the quartz plates of the round quartz cells and mounted in the IS center. IS_1_ is regularly calibrated with a calibrated light source (certified wavelength dependence of the spectral radiance), and the reliability of the setup calibration is then verified by measuring the emission spectra and Φ_f_ of certified spectral and Φ_f_ standards from Division Biophotonics.^30,39^

#### IS Setup IS_2_

IS setup Quantaurus 2 (C11347–11) from Hamamatsu is equipped with an 8 cm-diameter IS and has a fixed measurement geometry with a 53° angle between the excitation and detection pathways. Φ_f_ measurements of solid samples, such as powders or films, are performed in a quartz Petri dish (with or without a quartz lid) placed on the bottom of the IS. As the sample holder is part of the IS, an additional quartz Petri dish for the blank/sample is mandatory to avoid contamination of the IS surface. For correcting the measured emission spectra for the setup’s wavelength-dependent spectral responsivity, we utilized the emission correction curve implemented by the instrument manufacturer. The reliability of this correction curve was verified by determining the corrected emission spectra of the spectral fluorescence emission standard Kit BAM FCalKit, covering the wavelength range from 300 to 750 nm.^30,39^

### Φ_f_ Measurements

Room temperature Φ_f_ measurements consist of several steps: first, a measurement with a nonemissive blank in the IS is performed, which is then replaced by the luminescent sample measured under identical conditions as the blank. This includes the excitation wavelength and illumination geometry, detection range, integration time, and number of accumulations *N* (here *N* = 20). Here, all measurements were repeated for sample and blank for each side of the quartz cuvette (*N* = 4). The number of absorbed photons is then extracted from the difference between the intensities of the incident excitation light obtained for the blank and the sample and the number of emitted photons from the sample’s integrated luminescence spectrum.

### Transmission Spectroscopy

All transmission spectra were collected with a Lambda 1050 instrument from PerkinElmer.

### Scanning Electron Microscopy (SEM)

A Philips XL30 ESEM instrument was used to determine the average grain size of both the YAG:Ce and BaSO_4_ powders. The acceleration voltage was set to 30 keV. The average grain size was estimated from 20 grains from the respective secondary electron SEM image.

## Results and Discussion

To explore and quantify the influence of measurement geometry, sample positioning, and the optical properties of the blank on absolute Φ_f_ measurements of thin scattering, luminescent films, we first prepared 500 μm-thick polyurethane films containing different concentrations (1 wt % to 10 wt %, see the Experimental Section) of YAG:Ce microparticles. Also, a transparent polyurethane film was produced, and a series of polyurethane films containing various concentrations of scattering BaSO_4_ microparticles as blanks was adapted to the scattering properties of the luminescent film samples, all with a constant thickness of 500 μm. The film thickness was confirmed with a caliper (Table S1) and the homogeneity of the luminophore distribution in the films was verified by fluorescence measurements under 455 nm LED excitation (Figure S1).

**Figure 1:**
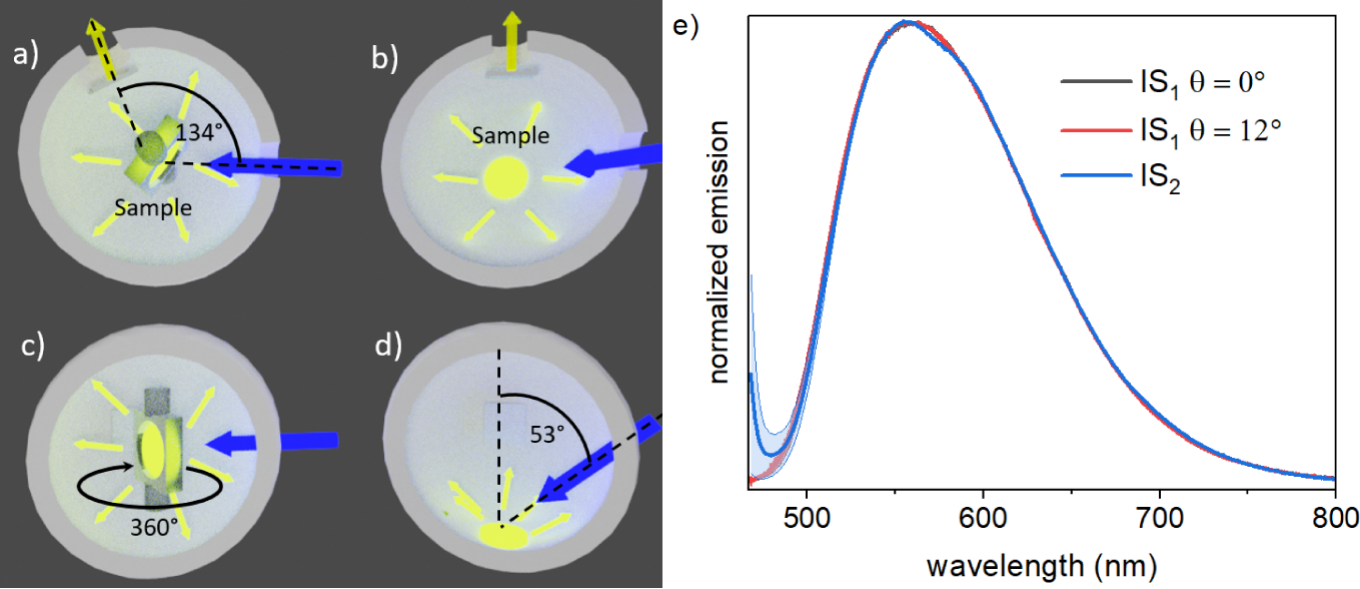
Top (a,b) and side (c,d) views of the excitation and detection pathways utilized in the IS setups IS_1_ (a,c) and IS_2_ (b,d). The main differences are (i) the excitation geometry, including the sample position and excitation angle, (ii) the collection geometry, (iii) the size of the IS, and (iv) the wavelength-dependent reflection of the IS coating material (Table S2). (e) Normalized emission spectra of the YAG:Ce microparticles embedded in the polymer film averaged over the YAG:Ce concentration series (1–10 wt %, uncertainty band given in light blue) collected with IS_1_ at θ = 0°(black) and θ = 12°(red) and with IS_2_ (blue). A comparison of the measured emission spectra reveals that the sample rotation in IS_1_ and the different excitation and detection geometries of both IS setups do not affect the spectral position, shape, or width of the YAG:Ce luminescence spectra.

Φ_f_ of the YAG:Ce-containing polymer films were absolutely measured with IS setups IS_1_ and IS_2_. The main differences between both IS setups are summarized in more detail in Table S2. For IS_1_, the samples were mounted in the center of the IS using a custom-designed rotatable holder, which allows for the realization of different sample illumination angles and, hence, different light distributions within the IS (see Figure 1a,c). For this study, the excitation light was perpendicularly focused onto the sample’s surface (excitation angle θ = 0°), thereby ensuring that the specular reflex from the sample left the IS. In addition, the samples were illuminated at an angle of θ = 12° to keep the specular sample reflex inside the IS. In IS_2_, the sample is placed in a quartz Petri dish at the bottom of the IS and illuminated at an angle of θ = 53° between the incident light and the sample surface. This is schematically shown in Figure 1b,d. The emission spectra of the polyurethane films containing YAG:Ce microparticles, recorded with IS_1_ and IS_2_, are displayed in Figure 1e. The comparison of the normalized emission spectra reveals that the illumination geometry, i.e., the angle between the incident excitation light and the sample surface, and the detection geometry do not affect the spectral position, shape, and width of the resulting YAG:Ce emission spectra.

### Influence of Illumination Geometry Using a Transparent Blank

**Figure 2:**
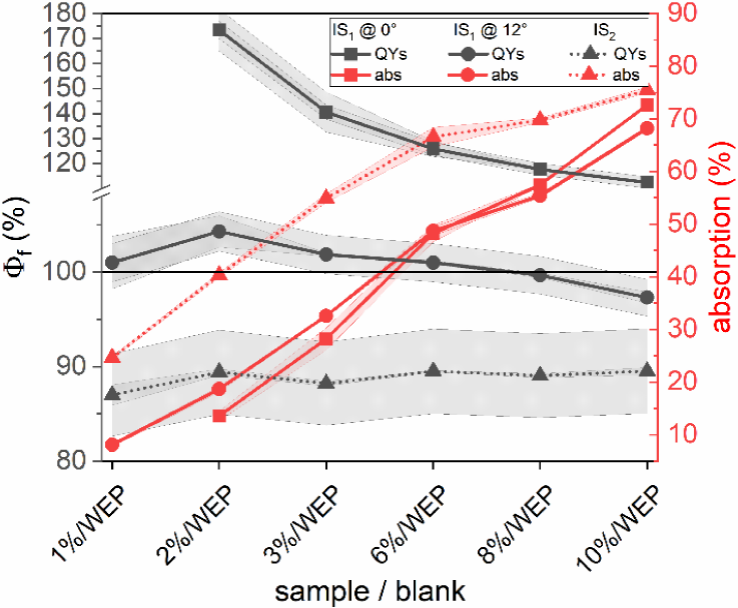
Absorption (red) and Φ_f_ (black) data of YAG:Ce microparticles embedded in a 500 μm-thick polyurethane film. The YAG:Ce amount was varied from 1 to 10 wt %. A transparent 500 μm-thick polyurethane film (“WEP”) was utilized as a blank. The measurements with IS_1_, using two different angles between the incident light and the sample surface (θ = 0°: squares; backreflected excitation light kept out of the IS; θ = 12°: circles; backreflected excitation light kept inside the IS), and IS_2_ (triangles) were performed at an excitation wavelength of 460 nm. The horizontal line indicates the physically meaningful limit of Φ_f_ of 100%.

First, the absorption and Φ_f_ of 500 μm-thick polyurethane films containing YAG:Ce powder in the concentration range of 1 wt % to 10 wt % were determined with IS_1_ and IS_2_ employing a 500 μm-thick transparent polyurethane film as the blank. For IS_1_, the sample-blank pairs were illuminated under two different angles (θ = 0° and θ = 12°), and for IS_2_, with the predefined angle of 53°. The results shown in Figure 2 reveal the expected increase in absorption with increasing YAG:Ce concentration. Generally, the absorption values measured with IS_2_ exceed those obtained with IS_1_.

This finding is attributed to reflection- and geometry-related differences of the two IS setups, i.e., (i) the size of the Spectralon-coated IS, and (ii) the illumination geometry, including the sample position within the IS and the excitation angle. The Φ_f_ values, including their standard deviations shown in Figure 2, amount to an average Φ_polymerIS2_ = (88.9 ± 0.9)% for IS_2_ while the Φ_f_ data obtained with IS_1_ exceed 100%, with the highest value of 170% for 2 wt %. The Φ_f_ values obtained with IS_1_ considerably depend on the sample illumination, i.e., the angle of the incident excitation light onto the sample surface, and the YAG:Ce concentration of the sample, and hence its absorption and scattering properties. The θ = 12° configuration seems to be better suited, as indicated by the generally smaller Φ_f_ values, which only partially exceed 100%, and the closer match of these Φ_f_ data with the ones obtained with IS_2_. These findings suggest that the backscattered reflex of the incident excitation light plays a significant role in the accuracy and reliability of Φ_f_ measurements of thin scattering, luminescent films. The still overestimated Φ_f_ values obtained for the θ = 12° geometry of IS_1_ are attributed to differences in the light distribution in the IS for the scattering sample and the nonscattering blank, as is further discussed in the next section.

**Figure 3:**
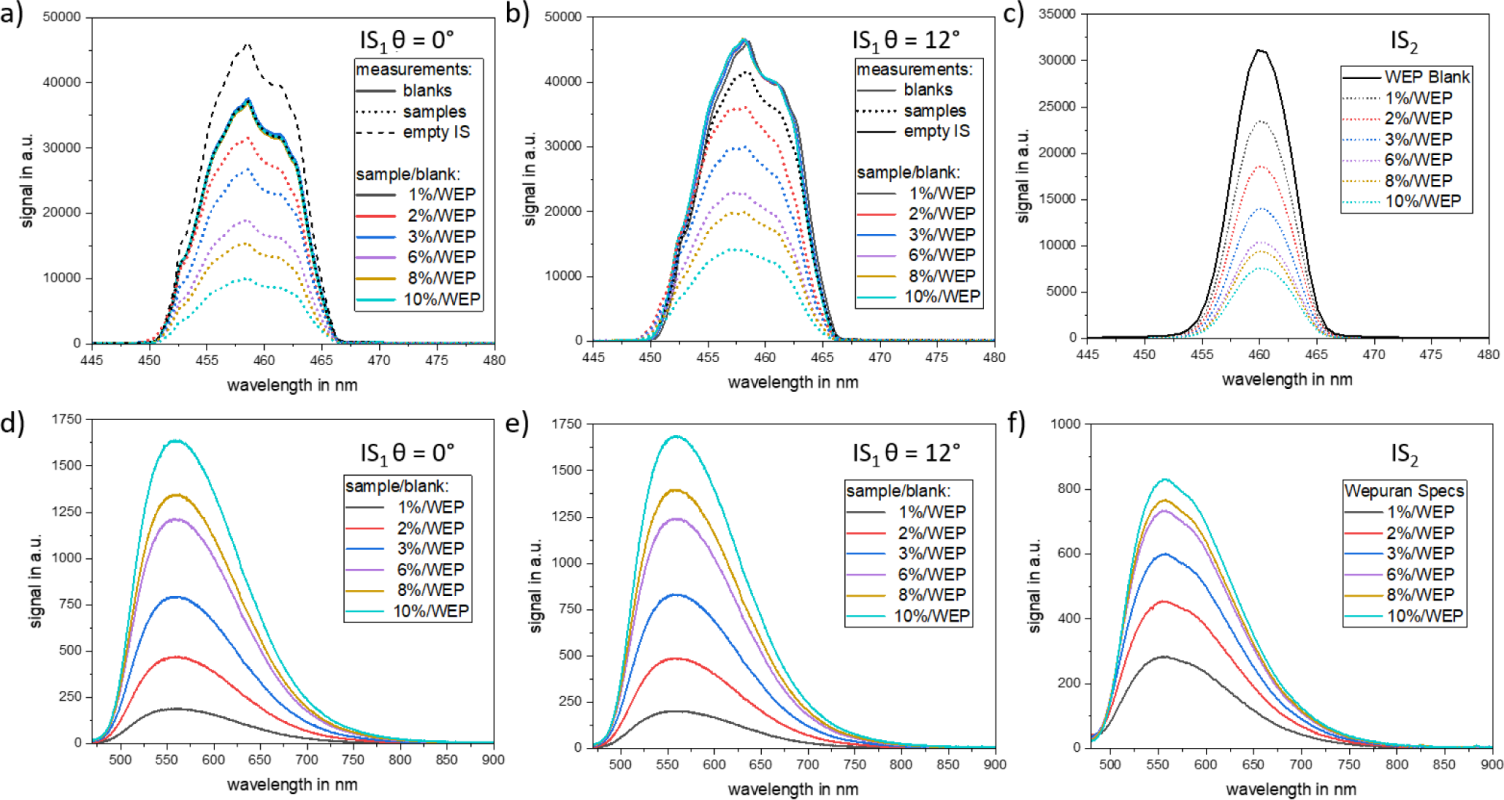
Excitation (a–c) and emission (d–f) spectra of the YAG:Ce concentration series in 500 μm-thick polyurethane films measured with IS_1_ using illumination geometries of θ = 0° (a,d) and θ = 12° (b,e) as well as with IS_2_ (c,f). The backscattered reflex is kept inside the IS for IS_1_ for θ = 12° and for IS_2_, but not for IS_1_ for θ = 0°. A transparent polyurethane film was used as a blank.

A closer examination of the two angles of incident illumination utilized for IS_1_ reveals that measurements at θ = 0°, where the backreflex leaves the IS, result in a reduced photon flux of about 24.5% for the blank/reference measurement compared to the empty IS. This follows from the comparison of the intensities of the corresponding excitation peaks shown in Figure 3a (solid lines vs dashed black line). This reduced photon flux originates from the reflection of the incident light at the different surfaces of the sample holder, i.e., the top and bottom surfaces of the quartz cuvette, air gaps between the quartz and blank material, and the polyurethane film. Using the Fresnel equations for normal incidence and the refractive indices *n* of the respective media (*n*_quartz_ = 1.55, *n*_polyurethane_ = 1.46), a total reflectivity of 24.2% is calculated. This value agrees well with the measured value of 25.6%. A comparison with the Φ_f_ measurements performed at θ = 12° shown in Figure 3b suggests that a sample rotation of 12°, where the backscattered reflex is kept inside the IS, can eliminate this effect. For this geometry, the intensities of the excitation peak are identical for the nonscattering polyurethane layer and the empty IS. This underlines that the backscattered reflex is essential for the reliable absolute determination of Φ_f_ of a scattering sample when using a transparent, nonscattering material as a blank. Nevertheless, this type of sample-blank combination is not recommended.

The emission spectra of the samples containing varying amounts of YAG:Ce microparticles are displayed in Figure 3d,e, indicating that the intensity and spectral shape of the YAG:Ce emission band are independent of the illumination angle. Figure 3a (θ = 0°) reveals that the film sample containing 1 wt % YAG:Ce seems to be nonabsorbing in the θ = 0° geometry, as its excitation spectrum (dashed black line) matches with the blank measurement (solid turquoise line). In contrast, the corresponding spectra of the 1 wt % YAG:Ce sample and the blank clearly differ for the θ = 12° illumination geometry (see Figure 3b). The relative differences in absorption values, i.e., the differences between the absorption of the sample and blank obtained with both illumination geometries, are illustrated in Figure S3. For the polymer film with a YAG:Ce concentration of 2 wt %, the relative difference in the total absorbed photon flux amounts to 80%, while it is reduced to about 20% for the 10 wt % YAG:Ce sample (see also Figure 3). We attribute this reduced relative deviation in absorption between the θ = 0° and 12° illumination geometries to an increase in light absorption caused by the increased YAG:Ce concentration. An underestimation of the absorbed photon flux in the θ = 0° geometry thus seems to directly correlate with the observed overestimation of Φ_f_ and the drastic drop of the Φ_f_ values with increasing YAG:Ce concentration shown in Figure 2.

Interestingly, the excitation light is more efficiently absorbed by the film samples in IS_2_ than in IS_1_, where the sample is placed at the IS bottom. In this configuration, the excitation light illuminates the sample at an angle of 53°, which can be either directly absorbed by the sample or reflected from the bottom of the IS back into the sample, where it can then be eventually absorbed. This leads to an apparently longer optical path length and a higher overall absorption probability compared to IS_1_. This backscattering effect only applies to semitransparent samples, which do not scatter and/or absorb all incident light at once. In addition, the size of the Spectralon-coated IS of IS_2_ is smaller than that of IS_1_ (8 cm vs. 15 cm). This also favors more efficient light absorption by the samples in IS_2_.

In the following, we focus on the θ = 12° geometry, while all measurements performed at θ = 0° are presented in the Supporting Information.

### Influence of Illumination Geometry Using a Scattering Blank

**Figure 4:**
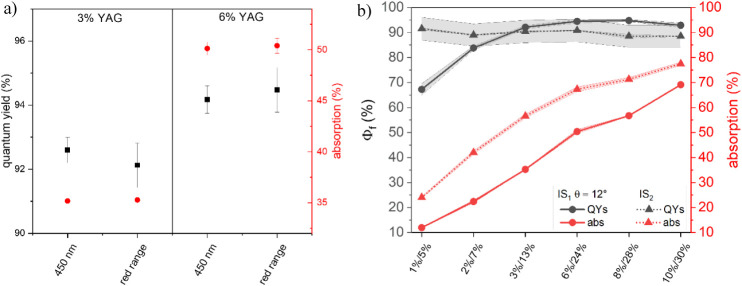
(a) Exemplarily chosen absorption (red) and Φ_f_ (black) values of the 500 μm-thick polyurethane films containing 3 and 6 wt % YAG:Ce measured with IS_1_ (θ = 12°) using scattering BaSO_4_-polyurethane blanks. For each sample, the BaSO_4_ concentration in the scattering blank was adjusted to the transmission spectrum of the corresponding luminescent film at 450 nm and in the red spectral range, where YAG:Ce is no longer absorbing (resulting in BaSO_4_ concentrations of 5, 7, 13, 24, 28, and 30 wt %). (b) Absorption (red) and Φ_f_ (black) values of the complete series of 500 μm-thick polyurethane films containing YAG:Ce concentrations of 1–10 wt % obtained with IS_1_ θ = 12°: circles) and IS_2_ (triangles). All Φ_f_ measurements were performed with an excitation wavelength of 460 nm.

To account for the scattering properties of the YAG:Ce samples and the light distribution in the IS caused by the scattering luminescent microparticles in the polymer films, we prepared polymer films containing small amounts of scattering BaSO_4_ microparticles as blanks. First, we determined the rough grain size of the YAG:Ce and BaSO_4_ powders using an SEM. As depicted in Figure S4, both materials show irregular particle shapes and a high polydispersity with an average size of (2.7 ± 0.8) μm and (1.3 ± 0.3) μm for the YAG:Ce and BaSO_4_ particles, respectively. To realize closely matching scattering properties of the sample and blank, the amount of BaSO_4_ added to the polyurethane films was chosen by matching the transmission properties of sample and blank (i) at the absorption maximum of YAG:Ce at 450 nm and (ii) in the red spectral range where YAG:Ce is not absorbing. The exemplarily chosen transmission spectra of the film samples containing 3 and 6 wt % YAG:Ce are shown in Figure S5. The corresponding absorption and Φ_f_ data, summarized in Figure 4a, reveal similar absorption and averaged Φ_f_ values for cases (i) and (ii). Compared to measurements using a transparent polyurethane film as a blank, the influence of sample illumination, i.e., the angle of incidence, on the resulting absorption and Φ_f_ values is considerably reduced for the scattering-adapted blanks, yielding Φ_f_ values below 100% within the experimentally derived standard deviations. The absorption and Φ_f_ data of the YAG:Ce concentration series measured with blanks of varying BaSO_4_ content with IS_1_ (θ = 12°) and IS_2_ are displayed in Figure 4b. The BaSO_4_ concentrations were selected according to case (ii). For IS_1_, an increased absorption was observed compared with the transparent polyurethane blank. For absorption values below 30%, the measured Φ_f_ increases with YAG:Ce concentration, while for larger absorption values (>30%), the Φ_f_ values are found to be independent of the YAG:Ce concentration. This points to a slight overestimation of the absorbed photon flux for low YAG:Ce concentrations and scattering blanks. However, when measurements with an absorption <30% are disregarded for IS_1_, an average Φ_f_ of Φ_BaSO4, 12deg_ = (93.4 ± 1.1)% is obtained (cf. Figure S6 for *q* = 0° geometry). Measurement with IS_2_ and the BaSO_4_-polymer films as blanks yield an averaged Φ_BaSO4IS2_ = (89.9 ± 1.2)% matching with Φ_polymerIS2_ = (88.9 ± 0.9)% obtained with the transparent polymer blank (cf. Figure 2).

As previously discussed, the back reflection of the incident light is negligible for a measurement geometry of θ = 12°; however, in the case of θ = 0°, 5 to 10% of the excitation light can still leave the IS due to reflections from the quartz surface of the cuvette, resulting in larger standard deviations for the Φ_f_ measurements (see Figures S7 and S8). In addition, diffuse scattering affects the distribution of the excitation light inside the IS. Depending on the scattering properties of the sample and the blank, the incident photons of the excitation light source are reflected from different surface areas within the IS, with the first reflection from the IS surface having the largest effect on the detected photon flux. The influence of the scattering blank on the measured absorption values is illustrated in Figure S8 by comparing the relative changes resulting from the presence or absence of scatterers in the blank.

For a small YAG:Ce concentration of 2 wt %, the absorption of the sample increased by 6% for a blank containing BaSO_4_ microparticles, while for the highest YAG:Ce concentration of 10 wt %, the relative absorption increase is below 2%. This also highlights the important role of the sample absorption at the excitation wavelength, which should be sufficiently high to minimize influences of the measurement geometry on the resulting Φ_f_ data.

### Influence of BaSO_4_ Concentration in the Scattering Blanks

**Figure 5:**
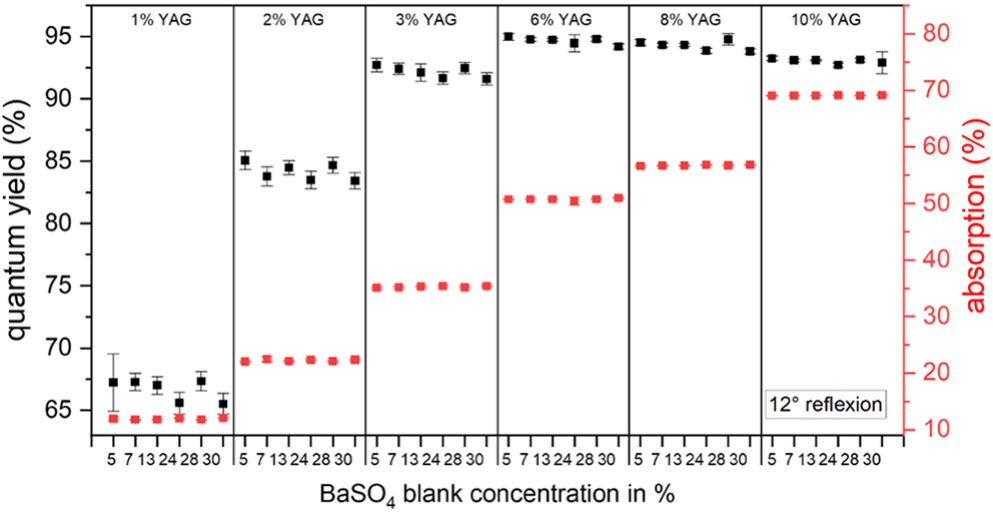
Φ_f_ of 500 μm-thick polyurethane films containing YAG:Ce concentrations of 1–10 wt % performed in IS_1_ at θ = 12° (black) and the corresponding absorption values (θ = 12°: red). For each luminescent sample, scattering blanks with BaSO_4_ concentrations ranging from 5 to 30 wt % were used, which were adapted to the sample transmission.

Lastly, we studied the influence of the number of BaSO_4_ scatterers in the blank on Φ_f_ measurements with IS_1_ (θ = 12°). The BaSO_4_ concentration series (5–30 wt %) was used as blanks for the entire YAG:Ce concentration series. As shown in Figure 5 (and in Figure S9 for θ = 0°), systematically underestimated Φ_f_ values are obtained for samples with small YAG:Ce concentrations such as 1 and 2 wt % due to excitation light losses and an overestimation of the absorbed photon flux caused by the different light distributions of the sample and blank within the IS. For absorption values of >30%, however, measurements with polyurethane blanks with low and high BaSO_4_ concentrations result in similar Φ_f_ values regardless of the angle of incidence of the excitation light. This finding suggests that while a scattering blank is necessary, the number of scatterers in the blank is not crucial to accurately and reliably determining Φ_f_ of such scattering luminescent films.

## Conclusion and Outlook

In this work, we explored, identified, and quantified typical sources of uncertainty in absolute fluorescence quantum yield (Φ_f_) measurements of scattering luminescent polymer films using integrating sphere (IS) spectroscopy performed with two typically used measurement geometries. Therefore, we systematically determined the Φ_f_ values of a series of 500 μm-thick polyurethane films containing varying amounts of scattering luminescent YAG:Ce microparticles with a custom-designed IS setup (IS_1_), enabling different illumination geometries, and a commercial IS setup (IS_2_) with a fixed measurement configuration. We thoroughly examined the following parameters: (i) the optical properties of the blank, using a 500 μm-thick transparent polymer film and polymer films containing different amounts of scattering BaSO_4_ microparticles, (ii) the sample position within the IS, (iii) the illumination geometry, i.e., the angle of the incident excitation light, and (iv) the detection geometry. For IS_1_, we exemplarily compared the two physically limiting cases of angles of incidence, θ = 0° and θ = 12°, where the excitation light is backscattered in such a way that it can leave the IS (θ = 0°) or is kept within the IS (θ = 12°). For IS_2_, the sample holder is part of the IS and, thus, sample orientation and illumination geometry are fixed to an angle of θ = 53°. Hence, the backscattered light remains in the IS. Our Φ_f_ measurements revealed that systematic errors originate from (i) setup configurations with center-mounted scattering samples where the reflexes from the sample can leave the IS due to diffuse backscattering or direct backreflection of the excitation light and (ii) the usage of a solid transparent blank for scattering luminescent film samples. The resulting uncertainties in Φ_f_ are particularly pronounced for thin, strongly scattering samples. (iii) The number of interfaces which cause reflections should be minimized and ideally be equal for the sample and blank. Although the angles of incidence are specific for the IS setups used in this study, the underlying physical concept is universally adaptable to both commercial and other custom-built IS systems with and without adjustable angles of incidence.

Based on the results of this study, we can provide users of different IS setups with information on how to interpret measured data and results and how the automatic data evaluation should be performed. For accurate and reliable Φ_f_ measurements, we recommend (i) a sample orientation that prevents backscattered and backreflected excitation light from leaving the IS, especially for a configuration with a center-mounted sample (cf. θ = 12° configuration of IS_1_), and (ii) a blank with scattering properties that closely resemble those of the sample. However, no exact match is needed, as indicated by the scatterer-independent Φ_f_ values determined for BaSO_4_ concentrations exceeding 5 wt %. This can differ for samples with higher or substantially lower scattering and absorption properties. Also, (iii) the absorption of the scattering luminescent samples must be sufficiently high. For example, for IS_1_, a critical value of 30% must be exceeded. This limit can be different for other IS setups as it depends on parameters like IS size and the wavelength-dependent reflectivity of the IS coating. (iv) For IS setups where the sample holder is part of the IS surface, as for IS_2_, contamination from absorbing and/or luminescent impurities introduced, e.g., by previously measured samples, can present a major source of uncertainty. To identify such effects, it is recommended to regularly monitor the IS for traces of contamination or aging by measuring the spectral response of the empty IS at typically used excitation wavelengths.

In the future, we plan to perform similar studies with different IS geometries for spectrofluorometers equipped with IS accessories and typical stand-alone IS setups, and compare the accordingly derived sources of uncertainties and requirements for accurate measurements.

## Supporting Information Available

Sample quality measurements, information about IS setups, Φ_f_ measurements and photon flux, powder grain size characterization, and optical properties of scattering films (PDF)
