# Supplementary material for: Influence of Measurement Geometry and Blank on Absolute Measurements of Photoluminescence Quantum Yields of Scattering Luminescent Films
Source: Anal Chem. 2025 Mar 7;97(10):5707–14. doi: 10.1021/acs.analchem.4c06726 (PMC11923943; doi:10.1021/acs.analchem.4c06726)
Supplement: Supplementary file 1 — ac4c06726_si_001.pdf [file ac4c06726_si_001.pdf]

Supporting Information
Influence of measurement geometry and blank on absolute measurements of photoluminescence quantum yields of scattering luminescent films

Florian Frenzel[+,a], Saskia Fiedler[+,a,b], Ahmad Bardan[a], Arne Güttler[a], Christian Würth[a], and Ute Resch-Genger[a*.]

[a] Division Biophotonics, Federal Institute for Materials Research and Testing (BAM), Richard-Willstaetter-Strasse 11, D-12489 Berlin, Germany. Email: ute.resch@bam.de.

[b] Photonic Materials, NWO-Institute AMOLF, Science Park 104, 1098 XG Amsterdam, The Netherlands.

Table of content

1. Sample quality measurements……………………………………………………………………………….... S2

2. Information about IS setups…………………………………………………………………………………………..S2

3. Φ_f – Measurements and photon flux………………....…………………………………………………………………................. S3

4. Powder grain size characterization…………………………………………………………………………….…... .S4

5. Optical properties of scattering films….……………………………………………………………………...……………S5

1. Sample quality measurements

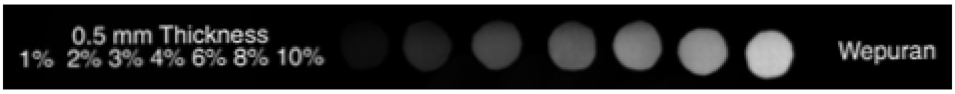

Figure S1 Luminescence homogeneity test of YAG:Ce embedded in polymer "Wepuran" and silicone with varying concentration and thickness. λ_{exc} = 455 nm.

Table S1 Nominal film thickness vs. measured thickness. The thickness of each sample was determined by averaging over three different positions of the sample. The uncertainty reflects only the statistical error.

Concentration (wt%)	Nominal thickness (mm)	Measured averaged thickness (mm)
1	0.5	0.47 ±0.02
2	0.5	0.48 ±0.01
3	0.5	0.48 ±0.01
4	0.5	0.49 ±0.01
6	0.5	0.49 ±0.01
8	0.5	0.49 ±0.01
10	0.5	0.50 ±0.01

2. Information about IS setups

Table S2 Differences of the two integrating sphere setups IS_1 and IS_2

Property/parameter	Home-built IS ("IS_1")	Quantaurus 2 (C11347-11) ("IS_2")
Light coupling	Fiber coupled	Free space
Sphere diameter	15 cm	8.38 cm
Sphere coating	Spectraflect	Spectralon
Detector unit	CCD (300 - 950 nm	CCD (300 - 950 nm)
Spectral resolution	0.1 nm	
Light source band-width		10 nm or less (FWHM)
Exc.-detec. angle	134-degree angle	28-degree angle
Long-neck cuvette<br>Position for solutions	Center mount,<br>Rotatable to exc. and collection	Center mount,<br>90-Degree to exc., 28-Degrees to collection
Laboratory dish<br>Position for solids	Center mount,<br>Rotatable to exc. and collection	Bottom,<br>Perpendicular to exc., 53-degrees to collection

The performance of an integrating sphere depends critically on its configuration, including numbers and sizes of ports, size of IS, baffle sizes and positions, and photon collection efficiency of the detection port. The main differences between both IS setups are summarized in Table S2. For IS_1, the excitation and detection channels are both fiber-coupled. The excitation channel consists of a single monochromator and a Xe lamp. Thus, the excitation spectrum exhibits a triangular shape due to the folding of the monochromator´s entrance and exit slit. The width and resulting line shape of such a triangle strongly depends on the width and shape of both slits. For commercial IS setup IS_2, which is completely housed, some geometries can be found in the corresponding patents such as a Xe lamp as excitation source, but more information is not available due to the company´s policy. This type of IS setup is increasingly used by industry/many companies and research groups and is employed here as a representative commercial IS setup.

3. Φ_f – Measurements and photon flux

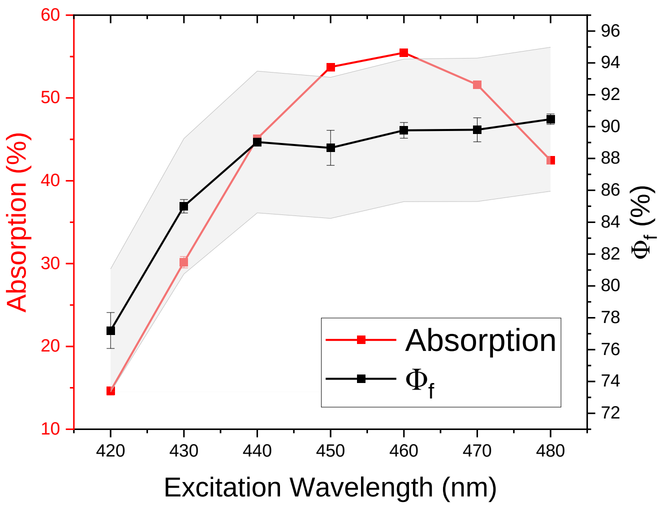

Figure S2 Absorption (red) and Φ_f (black) data of 3 wt% YAG:Ce embedded in a 500 µm thick polyurethane film obtained for varying the excitation wavelength from 420 nm to 480 nm in steps of 10 nm (10 nm increments). $BaSO_4$ powder embedded in a polymer matrix was used as a blank. Each measurement with IS_1 was performed in triplicate and the data were subsequently averaged. The error bars represent the statistical uncertainty, mainly from sample handling, while the gray shaded band represents the relative standard deviation of 5%.

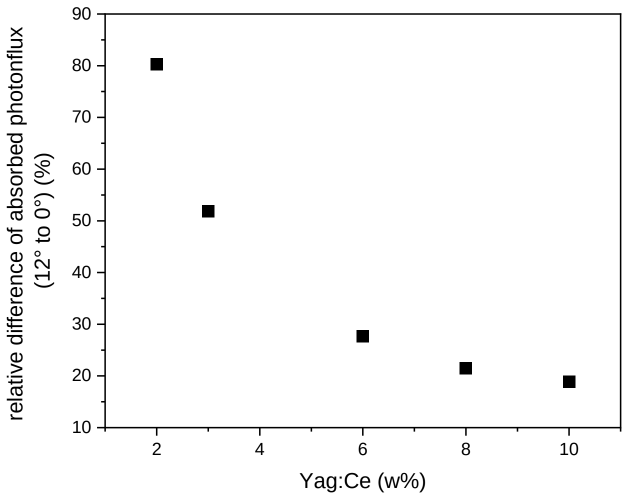

Figure S3 Difference of the absorbed photon flux between measurement geometries of θ = 12° and θ = 0° obtained with IS_1.

4. Powder grain size characterization

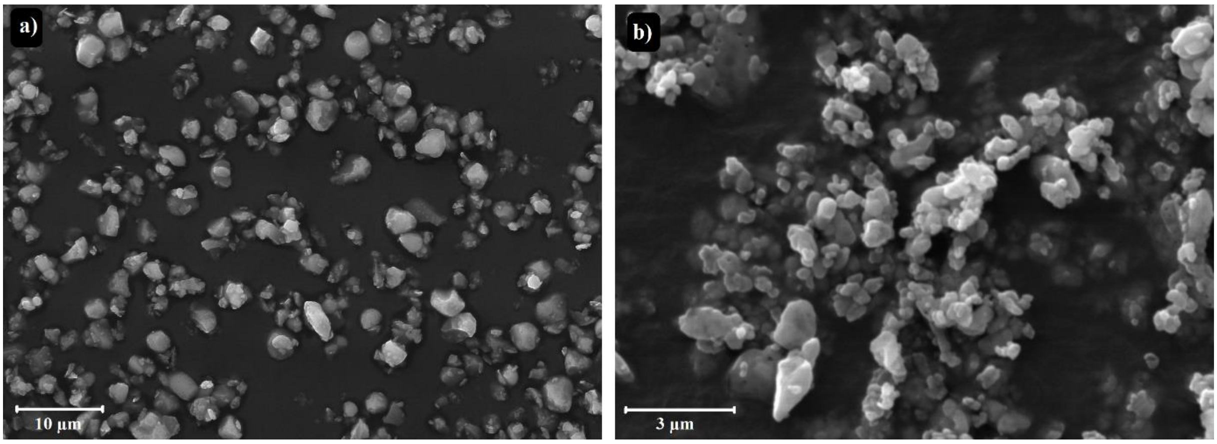

Figure S4: SEM images of (a) YAG:Ce powder and (b) $BaSO_4$ powder dispersed onto a clean Si substrate. YAG:Ce and $BaSO_4$ powders served as a raw material for the preparation of thin films acting as samples and blanks, respectively.

The grain size of each material was estimated by averaging over 20 grains in the secondary electron SEM images. Assuming a spherical shape, the average size of the YAG:Ce and $BaSO_4$ particles were determined to (2.7 ± 0.8) µm and (1.3 ± 0.3) µm, respectively.

5. Optical properties of scattering films

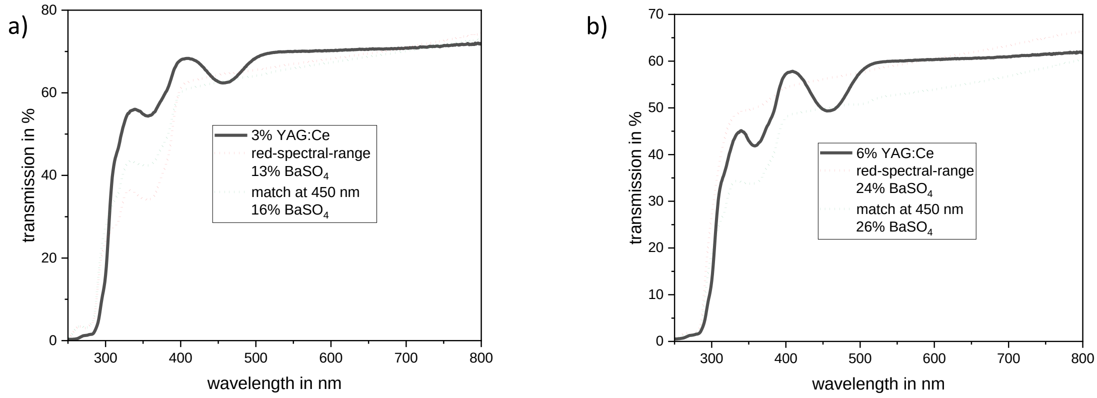

Figure S5 Transmission spectra of the 500 µm thick luminescent and scattering polyurethane films, used as samples and blanks used for Φ_f measurements, representatively done for films containing **(a)** YAG:Ce concentrations of 3 wt% and of 6 wt% (solid black lines) and **(b)** varying concentrations of $BaSO_4$ microparticles (dashed lines). The amount of $BaSO_4$ microparticles was chosen to match the transmission of the YAG:Ce thin films at 450 nm and in the red spectral range.

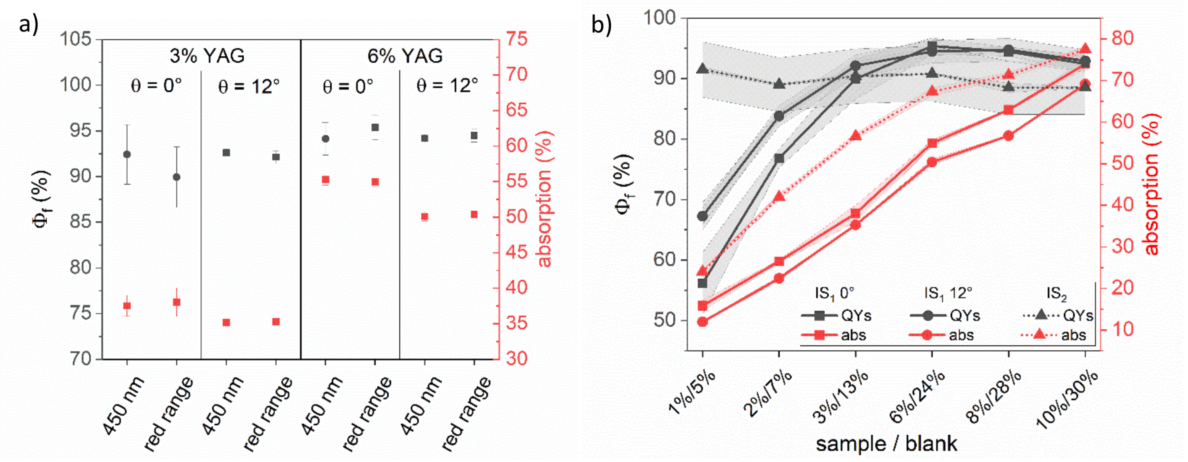

Figure S6 (a) Exemplarily chosen absorption (red) and Φ_f (black) values of the 500 µm-thick polyurethane films containing 3 wt% and 6 wt% YAG:Ce measured with IS_1 (θ = 0°: squares; θ = 12°: circles) using scattering $BaSO_4$-polyurethane blanks. For each sample, the $BaSO_4$ concentration in the scattering blank was adjusted to the transmission spectrum of the corresponding luminescent film at 450 nm and in the red spectral range, where YAG:Ce is no longer absorbent (resulting in $BaSO_4$ concentrations of 5 wt%, 7 wt%, 13 wt%, 24 wt%, 28 wt%, and 30 wt%). **(b)** Absorption (red) and Φ_f (black) values of the complete series of 500 µm-thick polyurethane films containing YAG:Ce concentrations of 1-10 wt% obtained with IS_1 (θ = 0°: squares; θ = 12°: circles) and IS_2 (triangles). All Φ_f measurements were performed with an excitation wavelength of 460 nm.

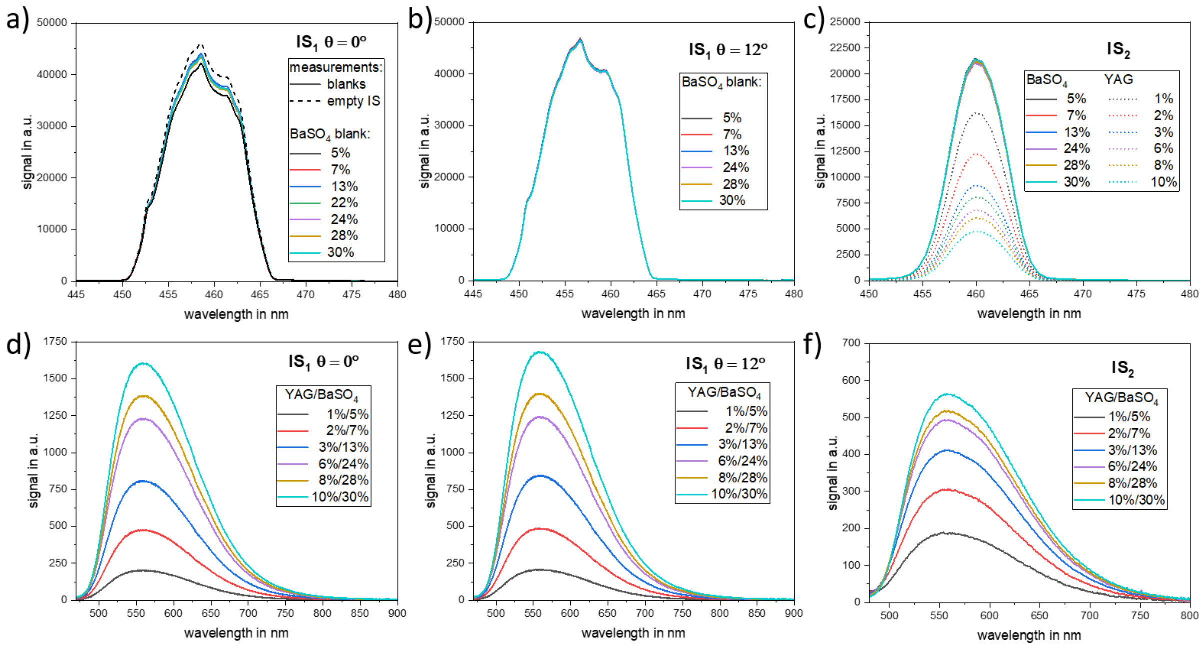

Figure S7 Excitation **(a, b, c)** and emission **(d, e, f)** spectra of the YAG:Ce concentration series measured with IS_1 using illumination geometries with θ of about 0°**(a, d)** and 12°**(b, e)** and with IS_2 **(c, f)**. The back-scattered reflex is kept inside the IS in IS_2 and IS_1, here only for θ of 12°, but not for θ of 0°. As blanks, 500 mm thick polyurethane films stained with $BaSO_4$ microparticles in concentrations of 5 w% to 30 w% were used.

For θ = 0°, a small amount of the excitation light still leaves the IS due to the reflection on the quartz surface of the cuvette. This results in a reduction of the overall incident photon flux by about 5 % to 10 % depending on the sample preparation and the number of (reflective) interfaces. Using the Fresnel equations for normal incidence and considering the reflection of all interfaces of sample and optical cell, we calculated a reflection of about10 % with an air gap between sample and quartz cuvette. A reflection of 5 % was determined for perfectly sealed sample and quartz cuvettes (no air gaps). Compared to the Φ_f measurements with a transparent blank, the amount of back-scattered light is considerably reduced as the incident light is rather diffusely scattered than directly back reflected. However, the averaged Φ_f value of $\Phi_{BaSO4,\ 0deg}$ = (92.7 ± 2.3) % has a slightly larger standard deviation than in the θ = 12° geometry ($\Phi_{BaSO4,\ 12deg}$ = (93.4 ± 1.1) %).

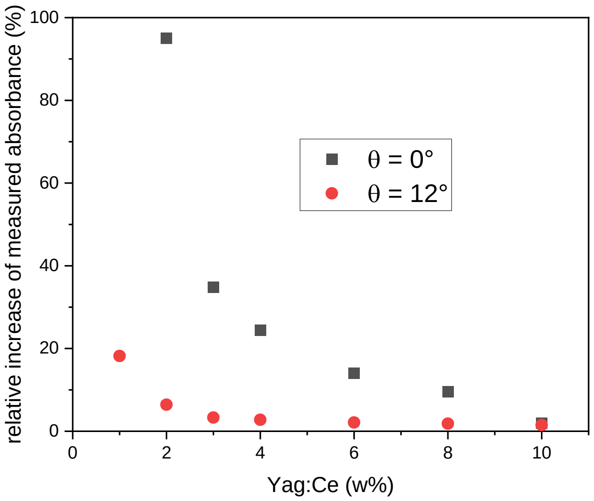

Figure S8 Difference of the absorbed photon flux obtained with IS_1 for measurement geometries of θ = 12° and θ = 0° with and without a scattering reference target.

For a small YAG:Ce concentration of 2 wt%, in the θ = 0° illumination geometry, the absorption of the sample increased by 95 % for a blank containing $BaSO_4$ microparticles, while for the θ = 12° configuration, only an increase of 6 % was observed. For the highest YAG:Ce concentration of 10 wt%, the relative absorption increase was below 2 % for both measurement geometries. This also highlights the important role of the sample absorption at the excitation wavelength which should be sufficiently high to minimize influences of the measurement geometry on the resulting Φ_f data.

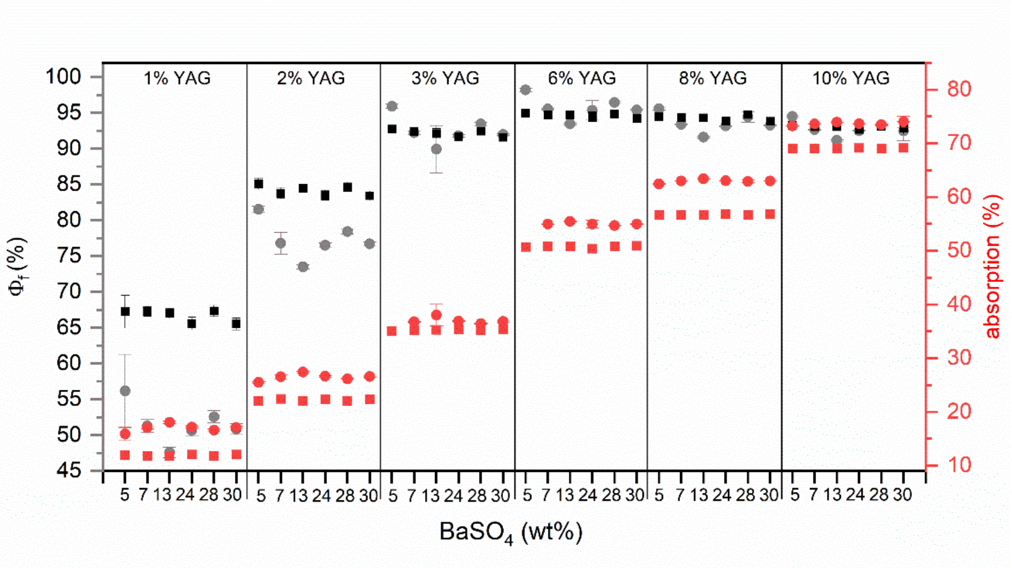

Figure S9 Φ_f of 500 µm-thick polyurethane films containing YAG:Ce concentrations of 1-10 wt% performed with IS_1 at θ = 0° (grey circles) and θ = 12° (black squares) and the corresponding absorption values (θ = 0°: red circles; θ = 12°: red squares). For each luminescent sample, scattering blanks with $BaSO_4$ concentration ranging from 5 wt% to 30 wt% were used, which were adapted to the sample transmission.
